# Wetting between Cassie–Baxter and Wenzel regimes: a cellular model approach

**DOI:** 10.1140/epje/s10189-021-00140-8

**Published:** 2021-11-16

**Authors:** Katarzyna Mądry, Waldemar Nowicki

**Affiliations:** Faculty of Chemistry, Adam Mickiewicz University in Poznań, Uniwersytetu Poznańskiego 8, 61-614 Poznań, Poland

## Abstract

**Abstract:**

The cellular model with periodic boundary conditions was proposed for the study of liquid–solid interface properties of solid surfaces decorated by a regular pattern. The solid surface was represented by a mosaic of truncated pyramids of two different slopes of side walls equivalent to a surface covered with triangular grooves of different dihedral angles. On the basis of the computations performed for a single elementary cell, the components of the interfacial energies and the apparent contact angles have been found for different Young contact angles and different tilting angles of the pyramid walls. It was found that at certain sets of angles, the wetting takes place with the partial coverage of the pyramid sidewalls—in between the Cassie–Baxter and Wenzel regimes. The influence of the line tension on the studied surface wettability was also examined.

**Graphic abstract:**

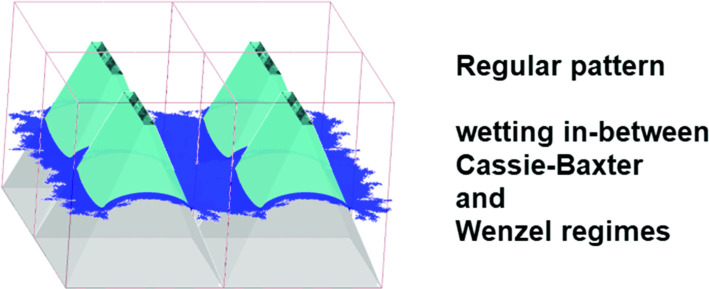

## Introduction

The hydrophobic surfaces decorated with micro- and nanoscale roughness are known to show useful and unique properties [1]. These properties have attracted a lot of interest due to their potential application in self-cleaning [2], anti-icing [3], anti-fouling [4], drag reduction [5], membranes for water–oil separation [6] to name a few. The origin of the properties is the specific way of wetting the surfaces—the liquid can wet only the upper parts of the surface irregularities, not filling the void between them and leaving an air layer underneath [5]. The mode of wetting in the conditions of reduced contact area between the liquid and the surface resembles the behavior of a water droplet settled on the surface of high hydrophobicity and negligible contact angle hysteresis [5]. The thermodynamic equilibrium contact angle $\theta _{\mathrm{Y}}$ on an ideally smooth and energetically homogenous solid surface is defined by the Young equation [7]:

1 $$\begin{aligned} \cos \left( {\theta _{\mathrm{Y}} } \right) =\frac{\gamma _{\mathrm{SV}} -\gamma _{\mathrm{SL}} }{\gamma _{\mathrm{LV}} } \end{aligned}$$

where $\gamma$ is the interfacial tension and indices SV, SL and LV relate to the solid–vapor, solid–liquid and liquid–vapor interfaces, respectively, while the “macroscopic” contact angle of the droplet settled on the heterogeneous surface is determined by details of surface inhomogeneities. One can imagine different ways of wetting. Some regular surface patterns allow the wetting of the surface elements in such a way that local surface fragments of solid surface are wetted at the Young angle and, at the same time, the free liquid surface is flat. Such a wetting at thermodynamic equilibrium can take place on some surfaces decorated by inhomogeneities with smoothly changing curvature or orientation of the local surface fragment to the plane parallel to the interface. Patterns at which such wetting occurs can be represented by the model of parallel cylinder used by Cassie and Baxter in research on porous surfaces [8] or the model based on the sine function presented by Johnson and Detree [9]. However, the model of rounded pillars studied by Bico et al. at $\theta _{\mathrm{Y}}>$ 90$^{\circ }$ leads to the convex inter-pillar liquid surface [10]. Similar results have been obtained by Afferrante and Carbone for conical pillars, hemispherical-topped cylindrical pillars and flat-topped cylindrical pillars [11].

When the irregularities on the surface contain sharp edges, the situation changes. For example, the surface decorated by cuboid pillars can be wetted in various ways. At $\theta _{\mathrm{Y}}=90^{\circ }$, the liquid can form a flat-free surface between walls of the cuboids at any elevation, but none of these states is thermodynamically stable since they do not correspond to the minimum energy. When $\theta _{\mathrm{Y}}>$ 90$^{\circ }$, the flat liquid surface should be stretched between the cuboid top edges, since in that case, the canthotaxis condition [12] is fulfilled, and the free liquid surface can be pinned to the edges resulting in the so-called “fakir effect” [13]. When $\theta _{\mathrm{Y}}<$ 90$^{\circ }$, the liquid wets the whole solid surface area under the droplet forming the Wenzel state [14]. So, the system can exist in one of two states defined by $\theta _{\mathrm{Y}}$. The same phenomena occur when the surface is covered by other polyhedra with flat faces and identical slopes of side face, e.g., pyramids, truncated pyramids, etc., however, at another $\theta _{\mathrm{Y}}$.

Assuming that on local inhomogeneities the Young law is still valid, side faces of pyramids may enforce the nonzero mean curvature of the liquid surface. In such a situation, especially, when the slopes of the pyramid faces are different, the wetting can be realized in different ways and its feasibility should be checked in thermodynamic categories. In practice, the flat liquid inter-pillar surface can be assumed for simplicity of calculation of the interfacial energy [4]. The assumption seems to be reasonable because of the possible small difference between the energy of flat and curved inter-pillar liquid surfaces. However, the assumption about the flat inter-pillar liquid surface is only an approximation, and to check its validity in all systems, the rigorous thermodynamic approach to the system should take into account all sets of assumptions about the shape of the liquid surface. The problem seems interesting to investigate in order to assess if the assumption of the surface curvature resulting from Young’s and Laplace laws can lead to the thermodynamically stable or metastable morphologies at some surfaces decorated with pillars and how the assumption may affect the results of model calculations.

Following the simplified classification of different regimes of wetting, convenient for the system decorated with truncated pyramids, based on Ref. [13], the apparent contact angle for the wetting occurring in the Cassie–Baxter regime (CBR, wetting the tops of truncated pyramids only) [8] is given by the Cassie law [15, 16]:

2 $$\begin{aligned} \cos \left( {\theta _{\mathrm{CBR}} } \right) =f\cos \left( {\theta _{\mathrm{Y}} } \right) -\left( {1-f} \right) \end{aligned}$$

where *f* stands for the fraction of liquid/solid interface area and 1—*f* is the fraction of liquid/vapor interface area. The $\theta _{Y}$ parameter stands for the Young contact angle (the contact angle for an ideally smooth flat surface).

When the liquid penetrates the whole surface under the droplet ($f\rightarrow$1), the wetting occurs in the so-called Wenzel regime (WR) [14]. In such a case, when the surface irregularities are small as compared to the size of the droplet, the apparent contact angle is defined by the Wenzel law [13]:

3 $$\begin{aligned} \cos \left( {\theta _{\mathrm{WR}} } \right) =r\,\cos \left( {\theta _{\mathrm{Y}} } \right) \end{aligned}$$

where *r* is the ratio of the rough wetted surface area to the area of the projection of this surface onto the parallel plane—the Wenzel roughness parameter.

In the mixed wetting state [17, 18] (further referred to as the intermediate regime (IR)), when the sidewalls of surface inhomogeneities are partly wetted, the apparent contact angle is given by [17, 19]:

4 $$\begin{aligned} \cos \left( {\theta _{\mathrm{IR}} } \right) =r\,f\cos \left( {\theta _{\mathrm{Y}} } \right) -\left( {1-f} \right) \end{aligned}$$

Formally, Eq. (4) is an extension of Eq. (2) into which the roughness factor has been introduced [4, 20].

The stability of the droplet deposited onto surfaces in CBR is mainly a result of the pinning of the liquid surface to the sharp edges of the surface inhomogeneities. However, the system may not be in the thermodynamic equilibrium—it can be switched to IR or WR, when the pinning is broken and the liquid fills partially or completely all surface cavities. However, the mechanism of wetting, particularly regarding the morphology of surface inhomogeneities, seems to be not fundamentally understood even when the considerations are limited to a geometric model in which the inhomogeneities are represented by simple regular solids [21, 22]. Usually, the droplet behavior on the solid surface decorated with such regular objects like pillars, prisms or more generally by truncated pyramids can be reduced to two situations:

1. If the geometry of the system fulfills the inequality

5 $$\begin{aligned} 2\theta _{\mathrm{Y}} >180^{0}+\alpha \end{aligned}$$

where $\alpha$ is the dihedral angle of the groove between pyramids, the droplet cannot reach the bottom of the groove. It also cannot partly wet the sidewalls of the pyramid, so as a consequence, the wetting in CBR is observed. The only parameter which can be influenced by system requirements is the pinning angle which has to fulfill the canthotaxis condition [12].

2. When

6 $$\begin{aligned} 2\theta _{\mathrm{Y}} <180^{0}+\alpha , \end{aligned}$$

the droplet can wet the bottom of the groove and the wetting of the system switches to WR. Moreover, when the Concus-Finn rule [23, 24]

7 $$\begin{aligned} 2\theta _{\mathrm{Y}} <180^{0}-\alpha \end{aligned}$$

is satisfied, the droplet vanishes as a result of liquid spreading along grooves.

When

8 $$\begin{aligned} 2\theta _{\mathrm{Y}} =180^{0}+\alpha , \end{aligned}$$

which is extremely difficult to reach in real physical systems, the thermodynamic analysis shows that the system is in equilibrium (in the absence of external force fields) at all states characterized by the liquid table elevation from the groove bottom to the tops of the truncated pyramids [25–27]. All possible elevations have the same internal energies, so they can be treated as equivalent. From the geometrical point of view, the liquid surface is represented by the horizontal surface of the curvature equal to zero at all elevations. In such a situation, the smallest fluctuation of system parameters can switch the analyzed system to CBR or WR. The transformation seems to be irreversible since the revers processes requires additional work applied to the system (the work of detaching the surface from edges).

**Fig. 1:**
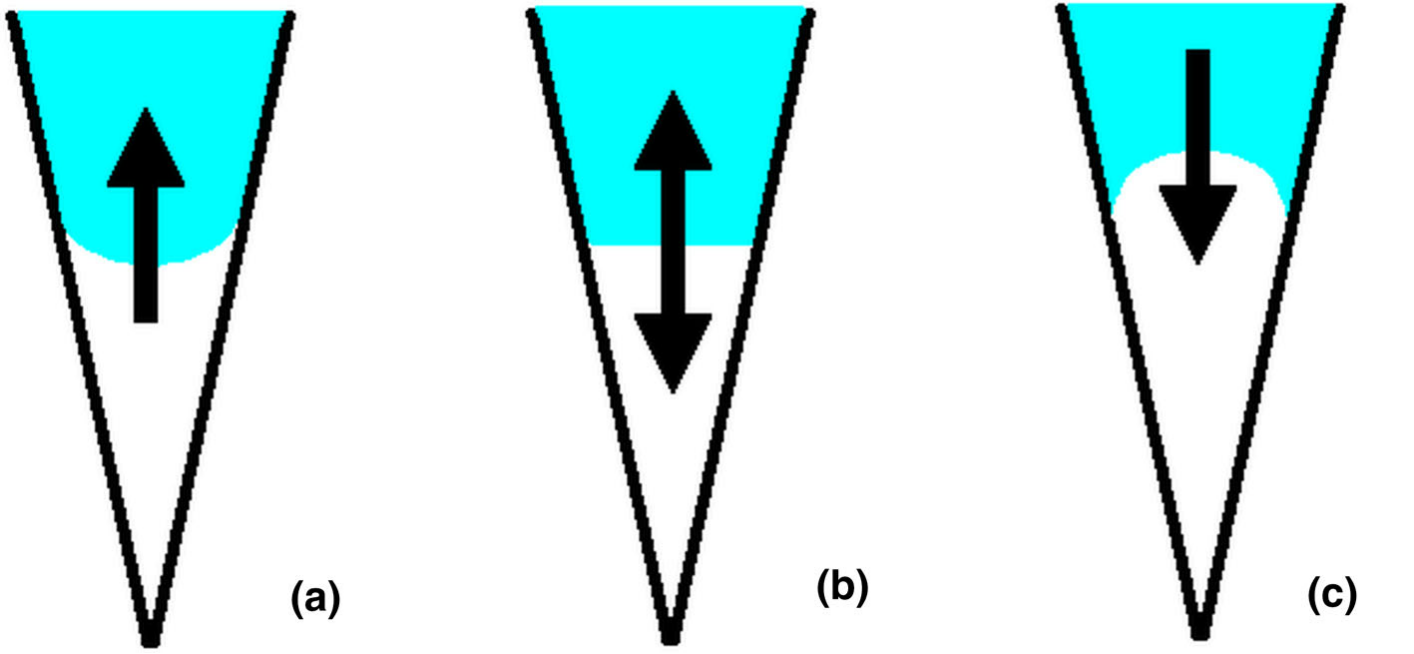
Tendency of liquid elevation in the wedge, corresponding to the requirements defined by formulas 5 **a**, 8 **b**, and 6 **c**

The morphology of the liquid surface in the wedge fulfilling the above requirements is shown in Fig. 1.

The preliminary calculations have shown that the situation diametrically changes when the slopes of sidewalls of truncated pyramids can take different values. In such a condition, the requirement 8 cannot be met simultaneously by two different groove angles and the system becomes able to switch to the stable state lying in between CBR and WR. The liquid surface, generally horizontal, gets nonzero curvature which has to satisfy the boundary conditions (contact angles) resulting from the Young law applied to the sidewalls of the studied surface irregularities, i.e., to walls of different slopes. As far as we know, no systematic studies on these surfaces have been published.

In the present study, an attempt was made to study the wetting phenomena of the surface decorated with truncated pyramids of different slopes of sidewalls and of the height of 10$^{-6}$ m forming a regular mosaic (see Fig. 3). The influence of geometrical details of the solid surface, as well as the Young contact angle and the liquid surface tension, are analyzed. Additionally, the impact of the line tension on the properties of the studied system is discussed since in the scale of irregularities applied ($< 10\ \upmu$m), one can expect a strong effect of the force acting along the triple contact line on the system behavior, especially taking into account that this triple line forms many closed curves around each pyramid, which results in a quite long total triple line under the whole droplet.

The study was performed through the energy optimization by the finite element method, using the Surface Evolver software (SE) [28, 29], by minimization of free interfacial energy of the liquid/solid and liquid/vapor interfaces at the bottom of the droplet. Since the numerical calculation for the whole system—the droplet and the solid surface—covered with hundreds or thousands of pyramids seems too complex and too much computation time-consuming, all calculations were performed for the periodic elementary cell of the surface containing only a single pyramid. Such a representation of the interface seems to be reasonable since the results can be extended to the wide range of droplet sizes (however, much larger then inhomogeneities) and the presence of gravity because both effects do not influence the apparent contact angle [11].

The paper is organized as follows: Firstly, the basic assumptions of the SE model applied to the study of the liquid/vapor and liquid/solid interfaces behavior are introduced (Sect. 2). Then, Sections: 3.1—the apparent contact angles and liquid surface elevation in grooves, 3.2—the groove dihedral angles and 3.3—the energies of wetting are presented in the search for the wetting regime between WR and CBR. Section 3.4 is devoted to the explanation of the formation of some intermediate regimes in categories of their energetic landscape. The effect of the line tension on the wettability is shown in Sect. 3.5. Finally, the conclusions drawn from the work are presented in Sect. 4.

## The model

**Fig. 2:**
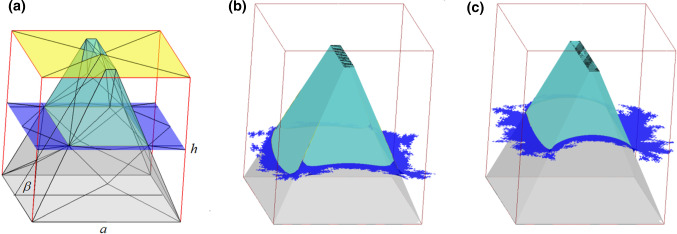
Initial structure of a single elementary unit cell used for calculations **a**. The yellow plane denotes the liquid surface in CBR, the blue plane—the initial liquid surface for IR optimization. **b** and **c** Show the result liquid surfaces obtained at $\theta _{\mathrm{Y}}=110^{\circ }$, $\alpha _{1}=30^{\circ }$, and $\alpha _{2}=30^{\circ }$ and $20^{\circ }$, respectively. The view is presented in the SE *raw_cells* mode

The simulation box is an elementary cell shown in detail in Fig. 2. The cell contains a truncated pyramid of the squared base located at the center of the cell. The geometry of the pyramid is characterized by its height *h* ($1.0\cdot 10^{-6}$ m), the length of the edge of its base *a* ($1.0\cdot 10^{-6}$ m), and by two slope angles of two opposite pairs of side walls $\beta _{1}$ and $\beta _{2}$. Consequently, the dihedral angles of the grooves are $\alpha _{1}$ and $\alpha _{2}$ (with $\alpha =2(90^{\circ } -\beta )$).

It is assumed that on the surface decorated with pyramids, a droplet of water with the volume of several to several dozen $\upmu$l is settled. Such a droplet is much larger than the heterogeneities on the solid support, i.e., it partly or completely wets a number of pyramids. The wetting is assumed to be reversible so the microscopic wetting hysteresis cannot occur. The applied computation cell represents a small repetitive fragment of the surface. There are no assumptions as to the thickness of the liquid layer above the cell, except for the effects of gravity to be negligible. The upper part of the cell is filled with water starting from the level of the liquid/vapor interface. The liquid/vapor interfacial tension is assumed to be equal to $\gamma _{\mathrm{LV}}=72.4\cdot 10^{-3}$ N/m. The Young contact angle on pyramid faces is $\theta _{\mathrm{Y}}$. The periodic boundaries at all walls of the simulation box are assumed (the torus model [28, 29]).

In the section devoted to the influence the line energy on the behavior of the droplet on pyramids, the positive line tension of the range $\sigma =0\, \div$ 6$\cdot$10$^{-8}$ N was applied [30]. The line tension was implemented as the energy of all edges mimicking the triple line (solid–liquid–vapor contact). In the model, these edges are of the *valence* equal to 3. (*Valence* is the internal SE variable that stands for the number of lattice facets adjacent to the edge).

The elements of the surface components are represented by a mesh of triangles. The SE program minimizes the free energy of the modeled system in defined steps including the procedures of mesh refinement, vertex averaging, polishing up the triangulation, and energy minimization [28, 29]. Mechanically stable interface configurations are obtained by minimizing the sums of all interfacial energies, which are functions of the coordinates of the nodes.

Minimization steps include a sequence of conjugated descents [28, 29]. At the end of most simulations, a couple of *hessian* commands (switching on the Newton method of free energy minimization) is executed. The final morphology of all interfaces in the simulation box consists of roughly 7000 individual nodes.

In most cases, the energy minimization started at the elevation of the liquid surface equal to half of the height of the truncated pyramid. Only part of the calculations devoted to the analysis of the energetics of reaching the equilibrium elevation by liquid surface was performed with the geometrical constraint consisting in the constant volume of the body, mimicking the air layer under droplet. This artificial assumption allowed the determination of the energy profile along the liquid elevation.

When the interfaces reached their final equilibrium morphology, some selected quantities were calculated employing internal variables and attributes of geometric elements of SE. These quantities include geometrical dimensions of the interfaces and their free surface/interface energies, elevations of liquid surface $h_{min}$, $h_{max}$, and so on. The free energies of interfaces are calculated as the sum of products of interface area and interface tension for each element of the lattice. The total free interface energy $E_{T}$ of the fragment of a droplet and solid located in the simulation box can then be expressed as

9 $$\begin{aligned} E_{\mathrm{T}} =E_{\mathrm{LV}} +E_{\mathrm{SL}} +E_{\mathrm{SV}} =\gamma _{\mathrm{LV}} A_{\mathrm{LV}} +\gamma _{\mathrm{SL}} A_{\mathrm{SL}} +\gamma _{\mathrm{SV}} A_{\mathrm{SV}} \end{aligned}$$

where $E_{\mathrm{LV}}$, $E_{\mathrm{LS}}$, and $E_{\mathrm{SV}}$ are the free energies, $A_{\mathrm{LV}}$, $A_{\mathrm{LS}}$, and $A_{\mathrm{SV}}$ are the areas of the liquid/vapor, liquid/solid, and solid/vapor interfaces, respectively. The $\gamma _{\mathrm{SV}}$ value was chosen arbitrarily as equal to 0.01 N/m. This constant is required by the assumed method of calculation. It influences all calculated energies as the integral constant but in the final results, this constant vanishes since the adhesion work and the contact angle do not depend on $\gamma _{\mathrm{SV}}$ and $\gamma _{\mathrm{SL}}$ individually but on the difference in these parameters. So, since the $\gamma _{\mathrm{SV}}$ can be defined as (from the Young equation 1):

10 $$\begin{aligned} \gamma _{\mathrm{SL}} =\gamma _{\mathrm{SV}} -\gamma _{\mathrm{LV}} \cos \left( {\theta _{\mathrm{Y}} } \right) \end{aligned}$$

the total free interfacial energy in the cell reads

11 $$\begin{aligned} E_{\mathrm{T}} =\left( {A_{\mathrm{LV}} -A_{\mathrm{SL}} \cos \left( {\theta _{\mathrm{Y}} } \right) } \right) \gamma _{\mathrm{LV}} +\left( {A_{S\mathrm{L}} +A_{\mathrm{SV}} } \right) \gamma _{\mathrm{SV}} \end{aligned}$$

Hence, the interfacial tension in the intermediate regime (IR) in between CBR and WR and the work of adhesion can be obtained as

12 $$\begin{aligned} \gamma _{\mathrm{SL}}^{\mathrm{IR}}= & {} \frac{\left( {A_{\mathrm{LV}} -A_{\mathrm{SL}} \cos \left( {\theta _{\mathrm{Y}} } \right) } \right) \gamma _{\mathrm{LV}} +\left( {A_{S\mathrm{L}} +A_{\mathrm{SV}} } \right) \gamma _{\mathrm{SV}} }{A_{\mathrm{C}} } \end{aligned}$$

13 $$\begin{aligned} W_{\mathrm{IR}}= & {} \gamma _{\mathrm{LV}} +\frac{A_{\mathrm{T}} }{A_{\mathrm{C}} }\gamma _{\mathrm{SV}} -\frac{E_{\mathrm{T}} }{A_{\mathrm{C}} }\nonumber \\= & {} \left( {1-\frac{A_{\mathrm{LV}} -A_{\mathrm{SL}} \cos \left( {\theta _{\mathrm{Y}} } \right) }{A_{\mathrm{C}} }} \right) \gamma _{\mathrm{LV}} \end{aligned}$$

where $A_{\mathrm{T}}$ is the total solid surface area and $A_{\mathrm{C}}$ is the surface area of the base of the pyramid.

Finally, the apparent contact angle of the analyzed system, i.e., of the liquid surface suspended in the void between truncated pyramids in IR is defined as

14 $$\begin{aligned} \theta _{\mathrm{IR}} =\mathrm{acos}\left( {\frac{A_{\mathrm{SL}} \cos \left( {\theta _{\mathrm{Y}} } \right) -A_{\mathrm{LV}} }{A_{\mathrm{C}} }} \right) \end{aligned}$$

where the surface area ratios A$_{\mathrm{LV}}$/A$_{\mathrm{C}}$ and A$_{\mathrm{SL}}$/A$_{\mathrm{C}}$ correspond to the fraction of liquid/vapor interface area *f*–1 and the product *rf* in Eq. (4), respectively.

The model $\theta _{\mathrm{IR}}$ parameter is the measurable quantity which gives the possibility to compare theoretical results with the experiment in the intermediate regime of wetting.

For comparison, let us notice that the wetting in the pure CBR or WR can be described by similar sets of equations written by the use of parameters of the model applied. In CBR, the pinning angle meeting the cantotaxis condition and the minimum of free liquid surface energy should be equal to $\beta _{1}$ and $\beta _{2}$, at two opposite pairs of sidewalls. In consequence, the surface is flat and the set of appropriate equations reads:

15 $$\begin{aligned}&\gamma _{\mathrm{SL}}^{\mathrm{CBR}} \nonumber \\&\quad =\frac{A_{\mathrm{upper}} \gamma _{\mathrm{SL}} +\left( {A_{\mathrm{T}} -A_{\mathrm{upper}} } \right) \gamma _{\mathrm{SV}} +\left( {A_{\mathrm{C}} -A_{\mathrm{upper}} } \right) \gamma _{\mathrm{LV}} }{A_{\mathrm{C}} } \end{aligned}$$

16 $$\begin{aligned}&W_{\mathrm{CBR}} \nonumber \\&\quad =\frac{A_{\mathrm{T}} }{A_{\mathrm{C}} }\gamma _{\mathrm{SV}} +\gamma _{\mathrm{LV}} -\gamma _{\mathrm{SL}}^{\mathrm{CBR}} \nonumber \\&\quad =\frac{A_{\mathrm{upper}} }{A_{\mathrm{C}} }\left( {1+\cos \left( {\theta _{\mathrm{Y}} } \right) } \right) \gamma _{\mathrm{LV}} \end{aligned}$$

17 $$\begin{aligned}&\theta _{\mathrm{CBR}}\nonumber \\&\quad =\mathrm{acos}\left( {\frac{A_{\mathrm{upper}} }{A_{\mathrm{C}} }\left( {1+\cos \left( {\theta _{\mathrm{Y}} } \right) } \right) -1} \right) \end{aligned}$$

where $A_{\mathrm{upper}}$ is the area of the rectangle truncating the pyramid. It should be noted that the ratio $A_{\mathrm{uppe}}$/$A_{\mathrm{C}}$ is equivalent to the fraction of liquid/solid interface *f* in Eq. (2).

**Fig. 3:**
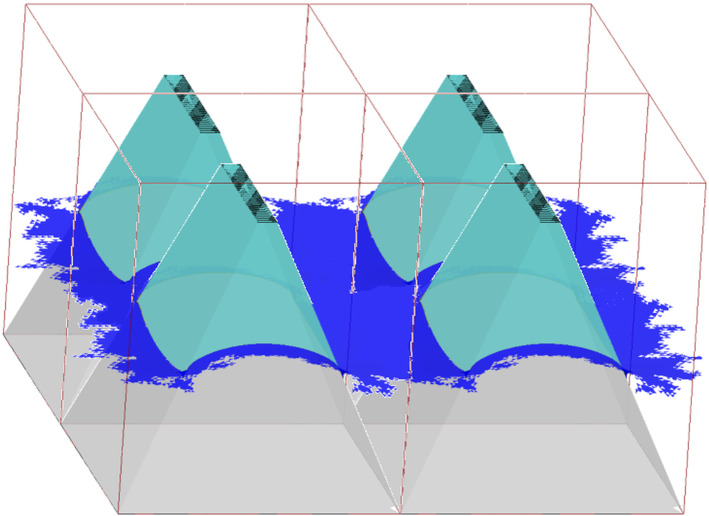
Fragment of the solid surface modeled by the set of four elementary cells from Fig. 2a

The contact angle for the wetting in WR can be calculated as follows:

18 $$\begin{aligned} \gamma _{\mathrm{SL}}^{\mathrm{WR}}= & {} \frac{A_{\mathrm{T}} }{A_{\mathrm{C}} }\gamma _{\mathrm{SL}} \end{aligned}$$

19 $$\begin{aligned} W_{\mathrm{WR}}= & {} \frac{A_{\mathrm{T}} }{A_{\mathrm{C}} }\gamma _{\mathrm{SV}} +\gamma _{\mathrm{LV}} -\gamma _{\mathrm{SL}}^{\mathrm{W}}\nonumber \\= & {} \frac{A_{\mathrm{C}} +A_{\mathrm{T}} \cos \left( {\theta _{\mathrm{Y}} } \right) }{A_{\mathrm{C}} }\gamma _{\mathrm{LV}} \end{aligned}$$

20 $$\begin{aligned} \theta _{\mathrm{WR}}= & {} \mathrm{acos}\left( {\frac{A_{\mathrm{T}} }{A_{\mathrm{C}} }\cos \left( {\theta _{\mathrm{Y}} } \right) } \right) \end{aligned}$$

where $A_{\mathrm{T}}$/$A_{\mathrm{C}}$ is equivalent to the roughness coefficient *r* in Eq. (3).

As mentioned above, Eqs. (3), (17), (3), (20), (4), and (14) are pairwise equivalent. In order to solve them, one needs to know the values of coefficients *r* and *f*. In the model presented, the calculation is trivial in the case of CBR and WR regimes but needs a numerical optimization method for the IR regime.

### Computations

The initial geometry of the cell containing a single truncated pyramid is shown in Figure 2a. The length of the pyramid base edge *a*, the pyramid height *h*, and one of the side slope angles $\beta$ are presented. The initial liquid surface is marked in blue. The exemplary final positions and shapes of the liquid surface after energy minimization at two different $\alpha _{2}$ values are shown in Fig. 2b and c. The yellow plane in Fig. 2a denotes the planar liquid surface formed in CBR.

As a result of boundary conditions applied to the unit cell, the presented model represents the infinite solid surface decorated by pyramids in the way presented in Fig. 3. Let us notice that the scattered triangles (shown using *raw_cells* SE option) from Fig. 2a complement each other composing a smooth liquid curved surface (accessible by *connected_cells* SE option) without blanks.

All computations were performed by means of minimization methods using the SE software, even in the case of quantities characterizing CBR and WR. Calculations for both regimes are easy to perform analytically (Eqs. 16, 17, 19 and 20) but the results obtained by SE allow tracking and check their convergence and credibility.

### System parameters

The behavior of the system studied depends on many parameters: the slopes of sidewalls of the pyramids, the size of their base, the liquid surface tension, the Young contact angle, to name a few. The part of parameters can be eliminated initially: the apparent contact angles resulting from surface inhomogeneity since it seems to be independent of the liquid surface tension (Eqs. 14, 17, and 20) and the scale of the geometrical dimensions of inhomogeneities (but for small values of the Bond [31] number only). However, the last independence seems to be risky since when the size scale falls in the $\upmu$m range, some additional effects such as the line tension should be taken into consideration. In the analysis presented below, the constant $\alpha _{1}=50^{\circ }$ arbitrarily selected was assumed.

**Fig. 4:**
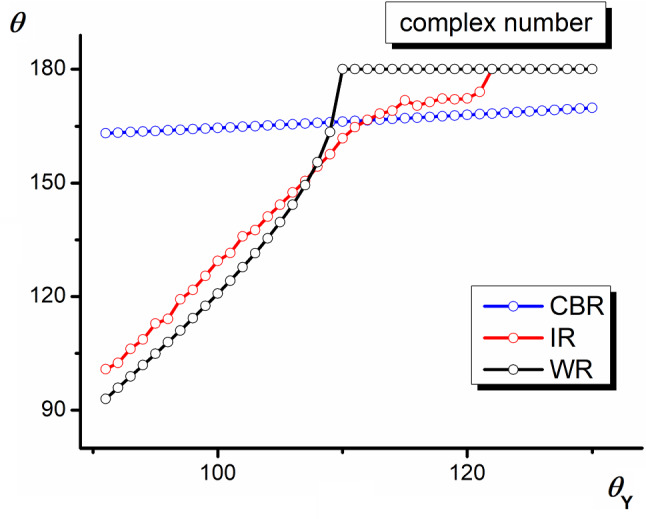
Dependence of the apparent contact angle on the Young angle in different wetting regimes ($\alpha _{1}=50^{\circ }$ and $\alpha _{2}=20^{\circ }$)

## Results

### Apparent contact angles vs. the Young angle

Figure 4 presents the apparent contact angles found for solid surfaces covered with pyramids with grooves characterized by $\alpha _{1}=50^{\circ }$ and $\alpha _{2}=20^{\circ }$ for all three wetting regimes. As expected, the apparent contact angles in the pure CBR take relatively high values and linearly increase with $\theta _{\mathrm{Y}}$ with a low regression coefficient. The apparent contact angles in WR increase much faster and nonlinearly with $\theta _{\mathrm{Y}}$. At a certain value (inequality 5), it reaches the real value 180$^{\circ }$ . For higher $\theta _{\mathrm{Y}}$ values, the apparent contact angle $\theta ^{\mathrm{WR}}$ becomes a complex number. It means that the wetting in WR is not possible at the thermodynamic equilibrium and the rough surface repulses the liquid trying to wet completely the whole surface of the grooves. In the wetting occurring in the intermediate regime (IR), the apparent contact angle increases similarly to the Young angle but taking values by $\sim 2^{\circ }$ higher. At a certain $\theta _{\mathrm{Y}}$ value, $\theta ^{\mathrm{IR}}$ takes a value smaller than $\theta _{\mathrm{WR}}$, crosses the $\theta _{\mathrm{CBR}}=f(\theta _{\mathrm{Y}})$ dependence, for a certain range of $\theta _{\mathrm{Y}}$, the values of $\theta ^{\mathrm{IR}}$ lie in between the WR and CBR curves, and finally $\theta ^{\mathrm{IR}}$ also assumes complex numbers.

**Fig. 5:**
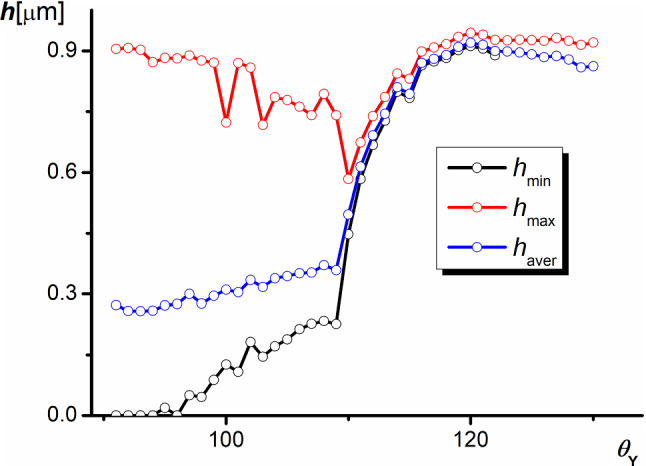
Dependence of the liquid surface elevation (the smallest elevation $h_{\mathrm{min}}$, the highest elevation $h_{\mathrm{max}}$, and the average elevation $h_{\mathrm{aver}})$ on the apparent contact angle of the Young angle in IR ($\alpha _{1}=50^{\circ }$, $\alpha _{2}=20^{\circ }$)

Figure 5 illustrates changes in the elevation of the liquid surface in the grooves in the case of IR. Generally, the liquid surface is curved with the same mean curvature at each point, satisfying the Pascal law. The level of the triple lines, as well as the elevation of the free liquid surface near the pyramid walls, depend on their slopes. In consequence, the elevation is widely distributed with a large difference between the altitude of the highest and the lowest point at small values of $\theta _{Y}$. For high $\theta _{Y}$ values, the liquid surface rises and flattens, which is associated with the reaching of the upper edge of the truncated pyramid close to CBR, which can be achieved at 1 $\upmu$m.

### Apparent contact angles vs. dihedral angles of the grooves

As mentioned above, the scale of the surface inhomogeneities does not influence the surface phenomena in the studied system provided that, on the one hand, the Bond number is small, on the other hand, the heterogeneities are so large that the line tension can be neglected. In such a situation, the only geometrical parameters controlling the wetting state are the groove dihedral angles $\alpha _{1}$ and $\alpha _{2}$.

**Fig. 6:**
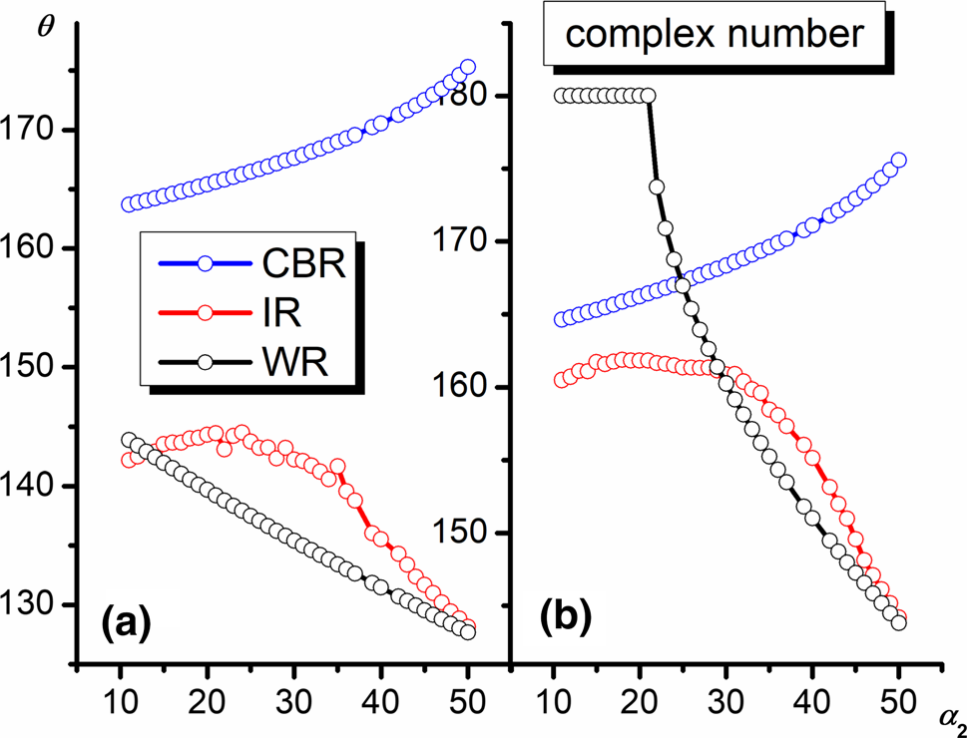
Dependence of the apparent contact angle on the dihedral groove angle $\alpha _{2}$ in different wetting regimes ($\theta _{\mathrm{Y}}=105^{\circ }$ **a**, $\theta _{\mathrm{Y}}=110^{\circ }$ **b**, $\alpha _{1}=50^{\circ }$)

Figure 6 shows dependencies of the apparent contact angle on the $\alpha _{2}$ value at a constant $\alpha _{1}=50^{\circ }$ and at two different Young contact angles 105$^{\circ }$ and 110$^{\circ }$, respectively. In both cases, the decrease in $\alpha _{2}$ causes a decrease in the apparent contact angle in CBR and its increase in WR. In the case of WR, the observed growth leads to the repulsion of the liquid since the contact angle becomes the complex number (Fig. 6b). The contact angles in IR behave similarly at both $\theta _{\mathrm{Y}}$ angles. They go through the maximum at $\alpha _{2}\approx$20$^{\circ }$ . However, the position of curves $\theta =f(\alpha _{2})$ in IR as well as those in CBR and WR changes significantly.

### The wetting energy

The apparent contact angles presented in the above section have physical meaning only when they correspond to the thermodynamic equilibrium of the system or if they represent the different local energy minima separated by energy barriers. In this section, the energy relations within the system are discussed in categories of the wetting energy *E* equivalent to the negative value of the work of adhesion *W*.

**Fig. 7:**
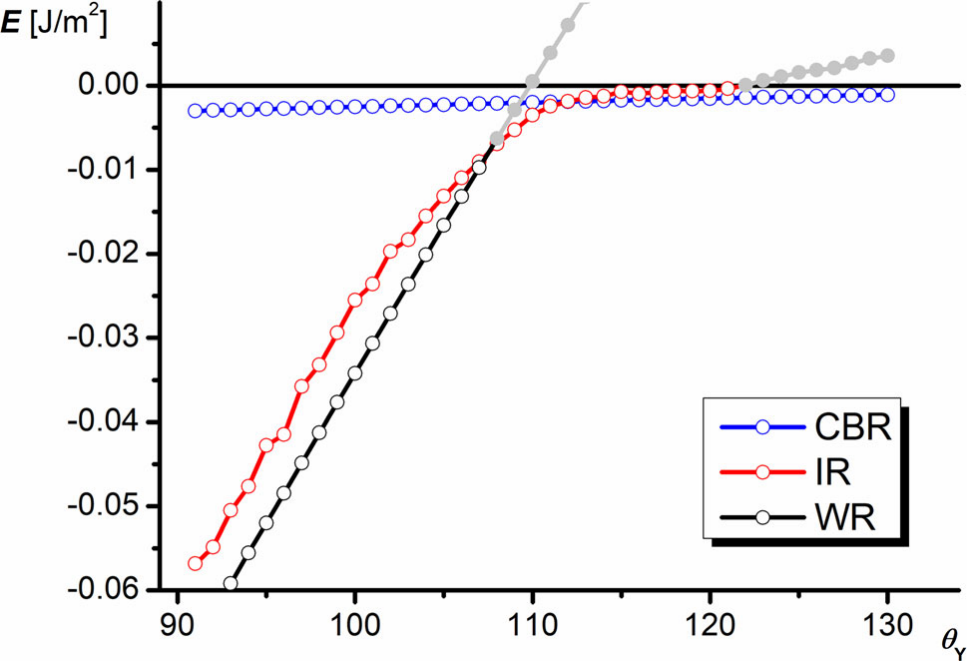
Dependence of the wetting energy on the Young angle in different wetting regimes ($\alpha _{1}=50^{\circ }$ and $\alpha _{2}=20^{\circ }$). The parts of curves in gray color do not have any physical meaning

Figure 7 shows wetting energies as a function of $\theta _{\mathrm{Y}}$ angle for the systems shown in Fig. 4. At the beginning of the analysis, let us notice that the system’s configuration at any CBR, even if its energy is higher than those of the others can be stable or metastable since it is always stabilized by the canthotaxis condition. This is not the case with WR since no special effects are stabilizing the structure, except the kinetic one which deals with the transportation of air along a solid surface. Nevertheless, one cannot expect a positive value of the wetting energy, so the positive parts of WR curves do not make any physical sense. The same applies to IR. Since WR is not stabilized by an energetic barrier as can be deduced from the thermodynamics, the system should switch to IR if the energy of IR is smaller than that of WR.

In consequence, for the system shown in Fig. 7, for relatively small values of $\theta _{\mathrm{Y}}$, the system is in WR or CBR, then it switches to IR or CBR at $\theta _{\mathrm{Y}}\approx$108$^{\circ }$ . In both cases, CBR corresponds to the local energy minimum. Starting at $\theta _{\mathrm{Y}}\approx 110^{\circ }$, the energy of IR takes values higher than that of CBR—so CBR becomes the thermodynamic equilibrium of the system. In the range $\theta _{\mathrm{Y}}>$ 122$^{\circ }$, the liquid at IR is repelled by the surface and the regime becomes unstable.

**Fig. 8:**
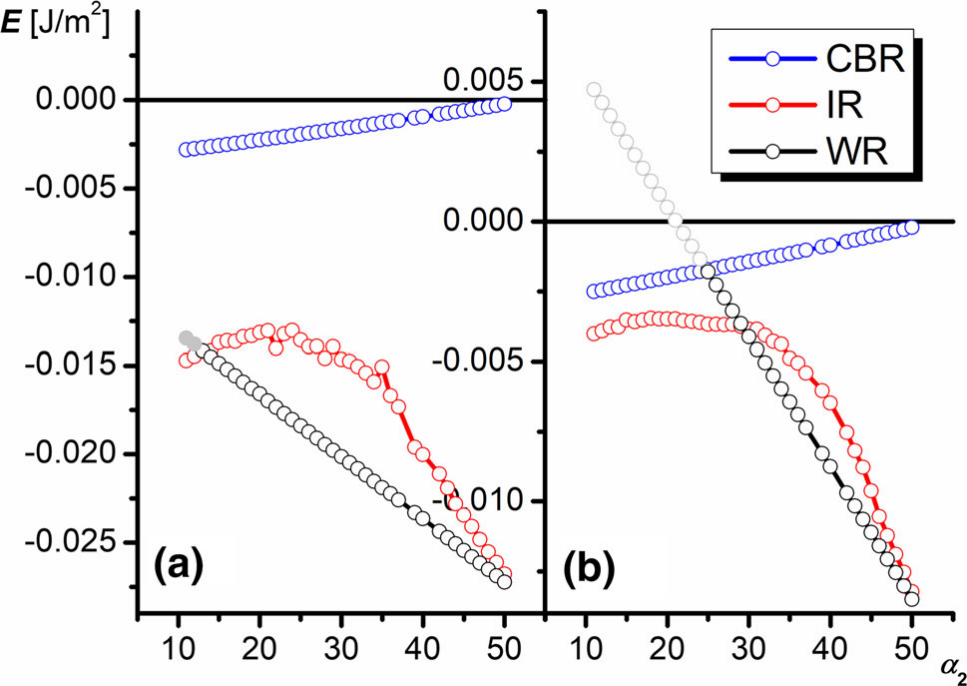
Dependence of the wetting energy on the dihedral groove angle $\alpha _{2}$ in different wetting regimes ($\theta _{\mathrm{Y}}=105^{\circ }$ **a**, $\theta _{\mathrm{Y}}=110^{\circ }$ **b**, $\alpha _{1}=50^{\circ }$)

Figure 8 presents dependencies of the wetting energy on the dihedral groove angle $\alpha _{2}$. At $\theta _{\mathrm{Y}}=105^{\circ }$ (Fig. 8a), the system practically in the whole studied range of $\alpha _{2}$ can exist in three states: the thermodynamically stable WR and metastable IR and CBR.

At $\theta _{\mathrm{Y}}=110^{\circ }$ (Fig. 8b), the increase in $\alpha _{2}$ causes successive states:

1. for $\alpha _{2}< 28^{\circ }$, the system can exist in CBR or IR, and IR is thermodynamically stable
2. for $\alpha _{2}> 38^{\circ }$, the system can exist in CBR, IR or WR, and WR is thermodynamically stable

At $\alpha _{2}>50^{\circ }$, the system switches to the single state—WR, since the energies of IR start to coincide with those of WR and the energy of CBR becomes positive.

### Profile of system energy along liquid elevation

The system’s behavior presented above, and in particular, the inhibition of switching between IR and the other regimes requires the existence of an energy barrier preventing the escape of the system from IR.

The real wetting regimes correspond to local or global minima of the surface/interface energy so they match the thermodynamic equilibrium or other metastable states. To predict the tendency of the system to the particular regime of wetting and answer the question whether the regime achieved by the system is the real thermodynamic equilibrium or the metastable state only, the wetting energy for different elevations of the liquid table is needed. Calculations of such energies were performed for the assumed constant volume of the body lying at the bottom of the grooves formed by parts of sidewalls of pyramids and bounded at the top by the liquid surface (see Fig. 2a). The body mimics the voids between pyramids filled with air. The wetting energies at CBR and WR, which are easy to obtain analytically, were also calculated by energy minimization for control purposes and comparison. The assumed gradually increasing constant volumes force the increase in elevation of the liquid table from the bottom to the top of the studied pyramids.

**Fig. 9:**
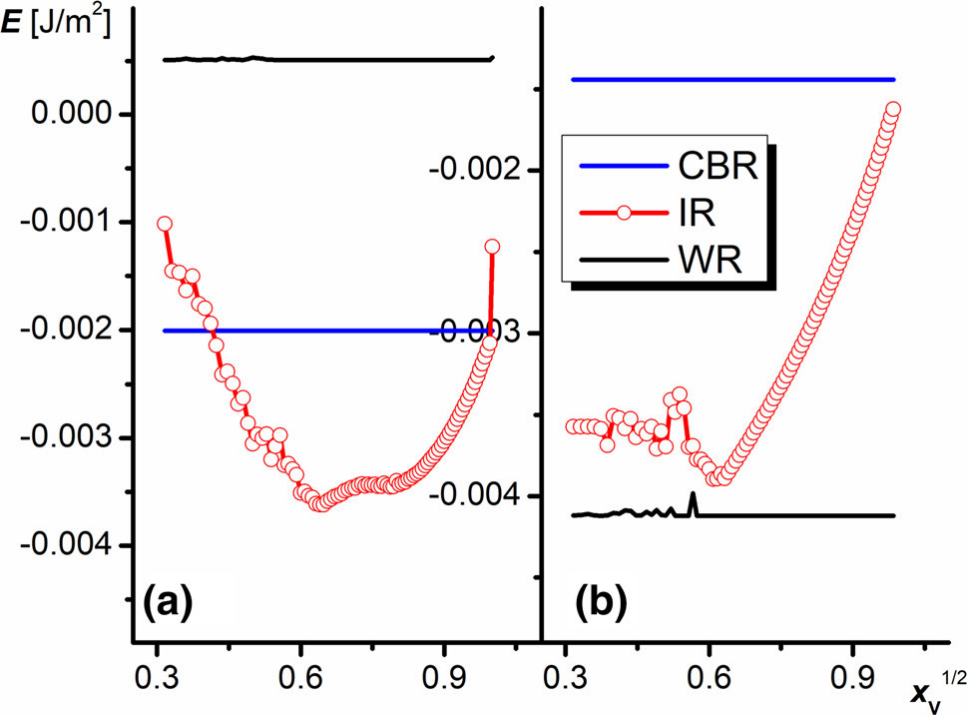
Dependence of the wetting energy on the $x_{\mathrm{V}}^{1/2}$ parameter for different wetting regimes ($\theta _{\mathrm{Y}}=110^{\circ }$ and $\alpha _{2}=20^{\circ }$ **a**, and $\alpha _{2}=30^{\circ }$ **b**)

Figure 9 presents the wetting energy (the negative work af adhesion) calculated for different square root volume fractions $x_{\mathrm{V}}^{1/2}$, defined as the ratio of the actual volume of the body filled with air to the volume of the body whose upper wall is the plane lying on the level of truncation of the pyramids (see Fig. 2a). The parameter $x_{\mathrm{V}}^{1/2}$ behaves similarly as the liquid surface elevation *h* presented in Fig. 5 (approximately $x_{\mathrm{V}}^{1/2}\sim h$ assuming the constant value of the liquid surface curvature). However, minimization of the system energy at a constant *h* does not make sense; hence, $x_{\mathrm{V}}$ was chosen instead. This procedure is convenient since the SE software gives the possibility of evolving the system at a constant volume. The calculations were performed for the constant values of dihedral groove angles $\alpha _{1}=50^{\circ }$, $\alpha _{2}=20^{\circ }$, and 30$^{\circ }$, and the contact angle $\theta _{\mathrm{Y}} =110^{\circ }$.

The curves $E=f(x_{\mathrm{V}}^{1/2})$ shown in Figure 9 represent the full energetic landscape of IR for different liquid elevation. The curves include minima corresponding to the potential well-surrounded by the energy barriers of the height comparable to the difference in energy between CBR and WR. The convergence of the results presented here is not very high, especially for small $x_{\mathrm{V}}^{1/2}$, but it is statistically significant. The minimum at $x_{\mathrm{V}}^{1/2}=0$ or 1 would correspond to WR or CBR. As shown, the energy minima are located at $x_{\mathrm{V}}^{1/2}\approx 0.58$ and 0.61 for $\alpha _{2}=20^{\circ }$ and $30^{\circ }$, respectively, indicating IR. For the first set of parameters (Fig. 9a), IR is equivalent to the thermodynamic equilibrium. At $\alpha _{2}=30^{\circ }$, the thermodynamic equilibrium is reached by WR, whereas IR is only metastable. It should be stressed here that the increase or the decrease in the liquid surface elevation in IR requires additional work since both minima are surrounded by potential barriers.

The curves $E=f(x_{\mathrm{V}}^{1/2})$ for IR shown in Fig. 9 illustrate the process of energy minimization, hence their minimums correspond to single points in Figs. 6b and 7.

**Fig. 10:**
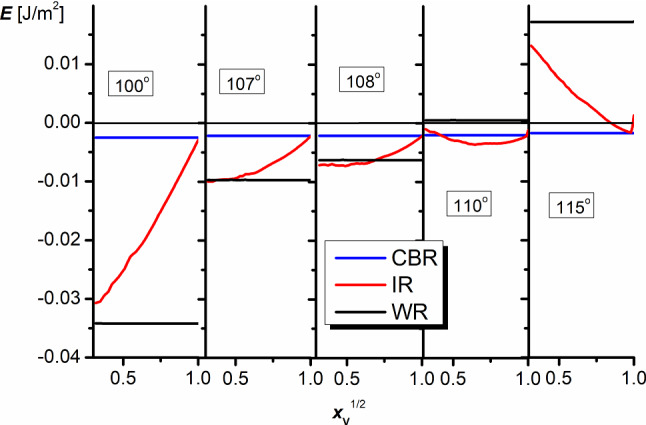
Dependence of the wetting energy at CBR, IR, and WR on the $x_{\mathrm{V}}^{1/2}$ parameter. The Young contact angles are marked in Figure ($\alpha _{1}=50^{\circ }$, $\alpha _{2}=20^{\circ }$)

Figure 10 presents the results of calculations of the system energy for different elevations of the liquid, forced by increasing air volume as $E=f(x_{\mathrm{V}}^{1/2})$, for dihedral groove angles $\alpha _{1}=50^{\circ }$ and $\alpha _{2}=20^{\circ }$, and the Young contact angles $\theta _{\mathrm{Y}}$ growing in small steps. As shown, the whole curves illustrating the wetting energies of IR obtained in the range of $\theta _{\mathrm{Y}}$ from 100$^{\circ }$ to 115$^{\circ }$ monotonically increase. An increase in $\theta _{\mathrm{Y}}$ also changes the shape of the curves. While the function is monotonically increasing when $\theta _{\mathrm{Y}}<$ 108$^{\circ }$, a minimum appears at higher $\theta _{\mathrm{Y}}$, then the minimum shifts to higher elevations. Finally, the system reaches the energy of CBR, and the system switches from IR to CBR. The observed phenomena in IR occur in the same $\theta _{\mathrm{Y}}$ range in which WR and CBR change takes place.

Of course, for each set of the system parameters, when the $E=f(x_{\mathrm{V}}^{1/2})$ dependence reaches a minimum and the energy in this state is smaller than WR and CBR, IR becomes thermodynamically stable.

For $\theta _{\mathrm{Y}}< 115^{\circ }$, the wetting in CBR corresponds to the metastable state which is stabilized only by the canthotaxis condition. The decrease in elevation requires some additional work to cover for the energy needed to deform the initially flat liquid surface. However, the CBR state can be easily broken by a fluctuation in energy. As a consequence, the system irreversibly reaches WR or IR corresponding to the global minimum of the system—to the equilibrium state.

Above $\theta _{\mathrm{Y}}>115^{\circ }$, the liquid, which tries to fill the grooves, is repulsed by the solid and the only regime available is CBR one.

In the region of 108$^{\circ }$–110$^{\circ }$, the system in the intermediate regime reaches the wetting energy smaller even than WR. In such a case, the numerical calculations suggest that the Young contact angle and dihedral angles of the grooves approximately fulfill the criterion:

21 $$\begin{aligned} 2\theta _{\mathrm{Y}} =180^{0}+\frac{\alpha _{1} +\alpha _{2} }{2} \end{aligned}$$

which seems to be a generalization of Eq. (8).

In the real system (when the liquid elevation can change freely), the system achieves thermodynamic equilibrium at the elevation spontaneously matching the minimum on the *E* vs. $x_{\mathrm{V}}^{1/2}$ dependence. In such a situation, the system can be observed in two states—at the global minimum in IR and the local minimum corresponding to CBR. Moreover, although it goes beyond the thermodynamic analysis which shows that the wetting energy of the state takes positive values, one might expect that the initially forced WR can be also metastable as a consequence of the fact that leaving this state requires the detachment of the liquid from solid and the delivery of air under the drop along the grooves on the solid surface.

**Fig. 11:**
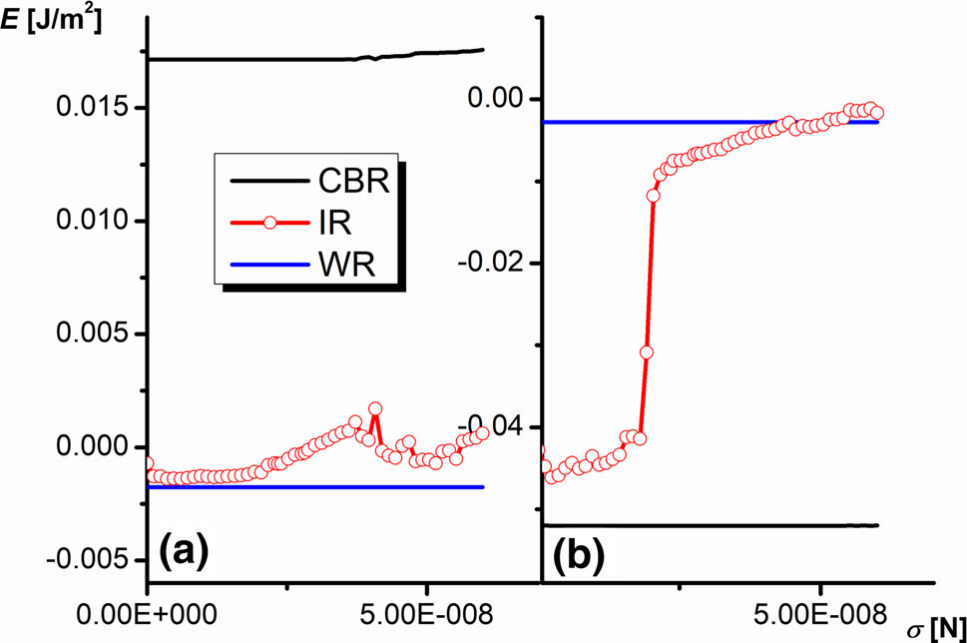
Dependence of the wetting energy on the line tension $\sigma$ at different wetting regimes ($\theta _{\mathrm{Y}}=115^{\circ }$ **a** and $\theta _{\mathrm{Y}}=95^{\circ }$ **b**, $\alpha _{1}=50^{\circ }$, $\alpha _{2}=20^{\circ }$)

In general, overcoming criterion 21 means that the thermodynamic equilibrium changes from the WR to IR as a result of the fact that the WR energy changes faster with $\theta _{\mathrm{Y}}$ than the CBR one. The switching is accompanied by appearance of stable intermediate regimes which can be observed in quite a wide range of Young angles.

### Line tension

One can expect that the wetting energy in WR is independent of the line tension $\sigma$ since there are no triple lines outside the perimeter of the drop. However, in the case of CBR and IR, the total length of the triple line under the drop is quite large since it is the sum of all perimeters of pyramids that decorate the surface and it is growing rapidly as the size of the prisms/triangular grooves decreases. While one can expect the linear dependence of the wetting energy on the line tension in the case of CBR, in IR, the line tension brings two contributions: the impact on the liquid surface level tending to the equilibrium state and the direct contribution of line energy to the free energy of the system.

Figure 11 illustrates both expected effects in the WR and CBR cases (for $\theta _{\mathrm{Y}}=95^{\circ }$ the expected linear dependence is invisible due to the small precision of the chart). However, for IR, the dependence seems to be more complex. For $\theta _{\mathrm{Y}}=115^{\circ }$, the superposition of the surface and line tensions produces two energy minima corresponding to $\sigma \approx$3.6 nN and 52 nN. For $\theta _{\mathrm{Y}}=95^{\circ }$, a dramatic increase in energy at a certain line tension $\sigma \approx$20 nN is observed suggesting the immediate switch of IR to WR (sliding the liquid surface to the bottom of the groove).

The results presented suggest that the line tension is an indisputable component of the free energy of the system even in the case of large drops.

## Conclusions

In contrast to the rough surfaces which can be characterized by equal slopes of all sidewalls of inhomogeneities, the surface decorated with grooves of different dihedral angle manifests the possibility to be wetted in the regime in between those of Cassie–Baxter and Wenzel, i.e., when the liquid wets only parts of the groove walls and the liquid surface is suspended between the tops and bottoms of the groove walls.

All three wetting regimes can compete to represent the local or global energy minima. The range of the Young contact angles at which partial wetting corresponds to thermodynamic equilibrium is relatively narrow. However, the partial wetting can correspond to the metastable or stable state coexisting with the Cassie–Baxter and/or Wenzel regimes.

For small Bond numbers, the regime choice and possibility of switching between regimes depend only on geometric details of the solid surface. However, even when the liquid drop is quite large, the line tension is capable of significantly changing the effective contact angle due to the large total length of the triple line on sidewalls of all pyramids. The effect is so large that can cause the switching from one regime to the other. Moreover, the effect gives the possibility of quite exact estimation of the line tension on the basis of measurements of the Young contact angle and the apparent contact angle on the grooved surface.
